# Gum Arabic Reduces C-Reactive Protein in Chronic Kidney Disease Patients without Affecting Urea or Indoxyl Sulfate Levels

**DOI:** 10.1155/2017/9501470

**Published:** 2017-05-14

**Authors:** Sarra Elamin, Mariam J. Alkhawaja, Amina Y. Bukhamsin, Mohamed A. S. Idris, Muntasir M. Abdelrahman, Nasrulla K. Abutaleb, Abdulrahman A. Housawi

**Affiliations:** King Fahad Specialist Hospital, Dammam 31444, Saudi Arabia

## Abstract

**Introduction:**

Gum Arabic (GA) is a complex polysaccharide with proven prebiotic properties and potentially beneficial systemic effects.

**Methods:**

We randomly allocated 36 chronic kidney disease (CKD) patients to receive 10, 20, or 40 grams daily of GA for four weeks and studied the systemic effects of this intervention.

**Results:**

Thirty participants completed the study with baseline glomerular filtration rate 29.1 ± 9.9 mL/min/1.7 m^2^. In contrast to previous observations, we found no effect on serum urea or creatinine levels. GA supplementation was associated with a small but statistically significant drop in serum sodium level (138 ± 2 to 136 ± 3 mmol/L, *p* = 0.002) without affecting other electrolytes, urine volume, or indoxyl sulfate (IS) levels. GA supplementation was also associated with a significant drop in C-reactive protein (CRP) level (3.5 ± 1.5 to 2.8 ± 1.6 ng/mL, *p* = 0.02) even in patients who received only 10 g/day (4.4 ± 1.2 to 3.2 ± 1.5 ng/mL, *p* = 0.03).

**Conclusions:**

Supplementing the diet of CKD patients with 10–40 g/day of GA significantly reduced CRP level which could have a positive impact on these patients' morbidity and mortality. This trial is registered with Saudi Clinical Trial Registry number 15011402.

## 1. Introduction

Chronic kidney disease (CKD) patients suffer from a persistent state of chronic inflammation and the gastrointestinal tract is a potential source for this inflammation. Uremia is associated with greatly increased bacterial counts in the duodenum and jejunum, higher prevalence of pathogenic bacteria in stool, and impaired intestinal barrier function [1].

The reasons behind this disruption of the intestinal microbiome are multifold. High concentrations of urea diffuse into the colon and provide a rich substrate for microbial multiplication. Impaired protein dissimilation in the small intestine allows significant amounts of protein to reach the colon, selectively encouraging the growth of proteolytic bacteria. Constipation allows more time for amino acid fermentation to occur in the colon and further encourages the growth of pathogenic bacteria [1].

The byproducts of amino acid fermentation diffuse from the colon into the circulation. Because of diminished renal clearance, their levels exhibit an exponential rise in patients with advanced renal insufficiency. Researchers have been focusing on two such uremic retention molecules; indoxyl sulfate (IS) and p-cresyl sulfate (PCS) [2]. Both toxins have been associated with renal function deterioration, cardiovascular disease, and overall mortality in the CKD population [3, 4].

Consequently, the intestinal microbiome is a potentially useful target for therapeutic interventions in CKD patients. Twenty years ago, Bliss et al. [5] demonstrated that supplementing the diet of CKD patients with a highly fermentable fiber (50 g/day) increased fecal bacteria mass by 50%, increased fecal nitrogen content by 41%, and reduced blood urea nitrogen (BUN) concentration by 12%. More recently, analyzing data from the National Health and Nutrition Examination Survey III demonstrated that high fiber intake was inversely related to C-reactive protein (CRP) levels and mortality in subjects with kidney disease [6]. This caused resurgence of researchers' interest in dietary manipulation of CKD patients.

Gum Arabic (GA) is a complex polysaccharide of natural plant origin [7]. It is a highly fermentable dietary fiber with proven prebiotic properties [8]. Beside the previously mentioned study by Bliss et al., there are other reports of nephrologists using it in economically disadvantaged settings to alleviate uremic symptoms and reduce the need for dialysis [9, 10]. It is also commonly used in folk medicine as a remedy for kidney disease.

The purpose of this study was to evaluate the short term systemic effects of GA supplementation in CKD patients. By promoting the multiplication of saccharolytic bacteria in the colon, we expected GA to suppress pathogenic bacteria and reduce the level of their toxic metabolites in blood. Consequent to this, we expected GA supplementation to improve inflammatory marker in CKD patients.

## 2. Methods

This was an open-label randomized clinical trial with parallel design. Our objective was to determine the effect of three different doses of GA on urea, uremic retention molecules, and inflammation in CKD patients. We estimated that 10 patients per group will give the study 80% power to detect a difference equal to one standard deviation in study parameters at the 0.05 significance level.

We recruited patients from the nephrology clinic at King Fahad Specialist Hospital in Dammam, Saudi Arabia. Ethical clearance was obtained from the institutional review board and all subjects signed informed consent forms before participation. Eligible candidates were adult CKD patients in stage 3B/4. Estimated glomerular filtration rate (eGFR) was calculated using the four variable MDRD formula. We excluded patients with malignancy, liver disease, intestinal resection, inflammatory bowel disease, recent antibiotic therapy, or recent GA use.

According to recruitment order, we divided patients into three equal groups using computer-generated random number lists. We gave each patient 28 labeled packages containing 10, 20, or 40 grams of GA in the form of instantly soluble granules (Agri-Rapid Acacia RE, Agrigum, UK). We asked patients to dissolve the contents of each package in a glass of water or juice and drink it daily for four weeks.

Two sets of data were collected, before and after the study intervention. This included clinical interviews to document the severity of gastrointestinal symptoms, fasting blood samples, and 24-hour urine collections. Routine laboratory investigations were performed on the same day using automatic analyzers. Frozen serum and urine aliquots were used to measure inflammatory markers and IS levels at a later date. CRP was measured using CRP Quantikine ELISA kits (R&B™, UK). Interleukins, transforming growth factor alpha (TNF-*α*), and interferon gamma (IFN-*γ*) were measured using Bio-Plex Precision Human Cytokine Assay (Biorad™, USA). Urinary transforming growth factor beta (TGF-*β*) was measured using TGF-*β* Quantikine ELISA kits (R&B, UK). IS was measured by liquid chromatography as described by Lin et al. [11].

SPSS version 19 was used for statistical analysis. Continuous and categorical variables were compared using paired *t*-test and marginal homogeneity test. *p* < 0.05 was considered statistically significant.

## 3. Results

**Figure 1:**
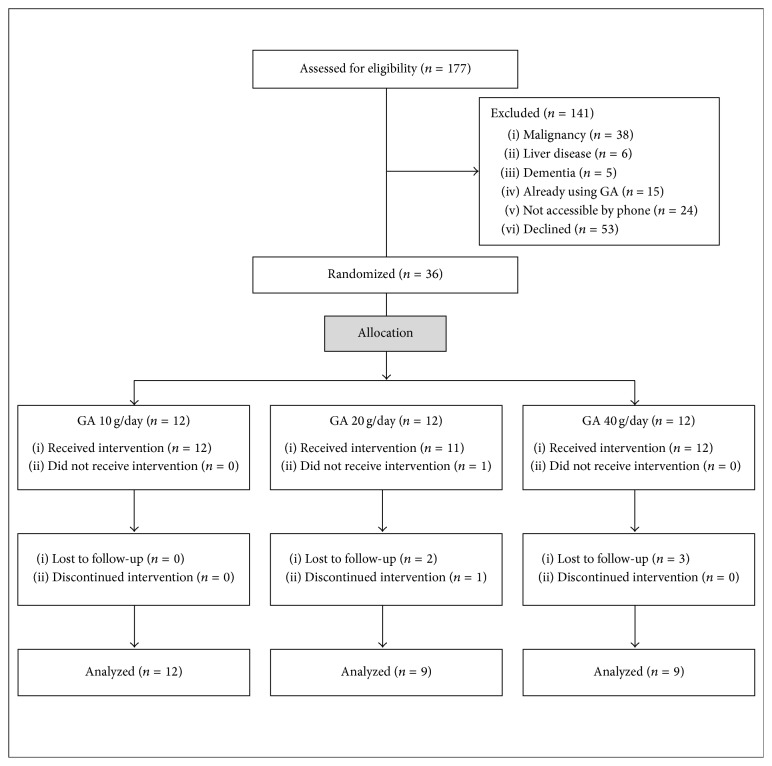
Consort flow diagram of clinical trial patients.

We screened all patients who were under regular follow-up at the nephrology clinic of King Fahad Specialist Hospital in Dammam. We identified 177 patients with CKD stage 3B/4, excluded 64 patients for various reasons, and were unable to reach another 24 patients. We invited 89 patients to participate in the trial; 53 patients declined and 36 patients consented to participate (Figure 1).

**Figure 2:**
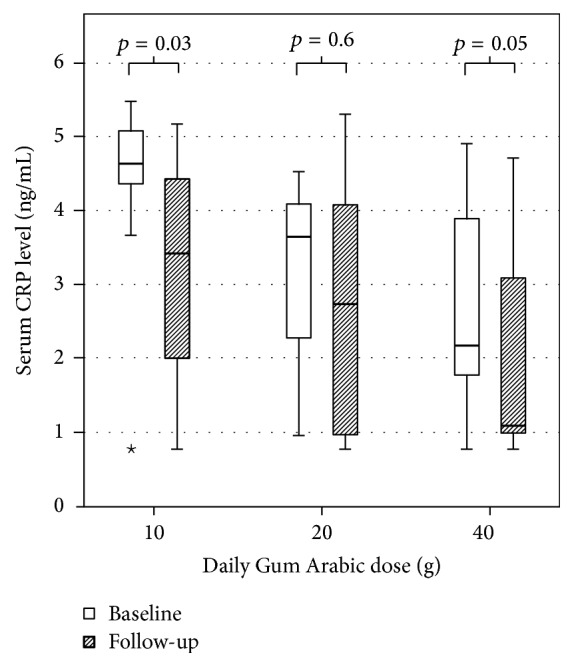
Baseline and follow-up C-reactive protein (CRP) values in different study groups. ^⋆^An outlier in the data displayed by this boxplot.

Thirty patients presented for follow-up and were included in the final analysis; their baseline characteristics are outlined in Table 1. After GA supplementation, there was a small but statistically significant reduction in serum sodium level (137.8 ± 2.4 to 136.3 ± 3.1 mmol/L, *p* = 0.002). There was no change in creatinine, blood urea nitrogen (BUN), potassium, calcium, magnesium, uric acid, parathyroid hormone, or hemoglobin levels. There was no change in urine volume, urea excretion, creatinine excretion, or protein excretion. IS levels were also not affected by the study intervention (Table 2). GA supplementation was associated with a statistically significant drop in CRP levels (3.5 ± 1.5 to 2.8 ± 1.6 ng/mL, *p* = 0.02), but there was no significant effect on other inflammatory markers, including urinary TGF-*β* (Table 3). The drop in CRP level was seen in all patient groups and was statistically significant in patients who received only 10 g/day (4.4 ± 1.2 to 3.2 ± 1.5 ng/mL, *p* = 0.03) (Figure 2).

GA supplements were well tolerated with no significant gastrointestinal side effects (Table 4). One patient discontinued the supplements after one dose because she did not like the texture of the solution. One patient returned 14 doses, three patients returned three doses, two patients returned two doses, five patients returned one dose, and nineteen patients returned no doses. Overall, 93% of dispensed doses were consumed.

## 4. Discussion

Dietary fibers are a heterogeneous group with variable physiochemical properties determined by the physical and chemical composition of the fiber. The physiological actions of a particular dietary fiber are determined by those properties, including susceptibility to bacterial fermentation, water-holding capacity, cation-exchange, and adsorptive functions [12]. This explains the different physiological responses obtained after dietary supplementation with different fibers.

During in vitro studies, GA demonstrated the highest short chain fatty acid (SCFA) production and organic matter disappearance by fermentation (69.5%) compared to soy fiber (56.4%), oat hull fiber, carboxymethyl-cellulose, and psyllium (all less than 20%) [13]. In another experiment, GA produced a greater prebiotic effect than an equivalent dose of inulin and had excellent tolerance [8]. Because of these observations, we expect GA to be the optimal dietary fiber supplement for patients with CKD.

In contrast to previous observations, GA supplements had no effect on BUN levels in this study. Initially high levels of nitrogenous waste products and strict adherence to a low protein diet may be necessary for dietary fiber supplementation to have an appreciable effect on BUN level. In the previously mentioned trial by Bliss et al. [5], patients had advanced renal insufficiency with baseline creatinine levels of 390 ± 70 *μ*mol/L compared to 225 ± 77 *μ*mol/L in our cohort. Previous reports of GA reducing BUN and creatinine levels were also obtained from patients with advanced renal insufficiency or dialysis dependency [9, 10]. As for other dietary fibers, supplementing the diet of CKD patients with arabinoxylan oligosaccharide (20 g/day) or pea hull (10 g/day) and inulin (15 g/day) also failed to reduce BUN levels [14, 15]. Supplementing the diet of HD patients with high-amylose corn starch (15 g/day) had no effect on BUN level [16] while oligofructose-enriched inulin (20 g/day) modestly reduced urea levels from 126 to 119 mg/dL [17].

Similarly, IS levels were not affected by GA supplementation in this trial. This could be explained by relatively low baseline levels indicating still efficient renal clearance. In a study designed to demonstrate the effect of an oral adsorbent on IS levels, the investigators selected patients with baseline creatinine clearance less than 20 mL/min and baseline IS levels greater than 5 mg/L [18]. In the current study, only two patients with advanced renal insufficiency had similarly high IS levels at baseline and both levels dropped after the study intervention (12.5 to 11.2 and 10.6 to 7.8 mg/L). Measuring 24-hour urinary excretion rates would probably reflect the magnitude of IS production by colonic bacteria more accurately than serum levels, especially in patients with moderate renal insufficiency. Unfortunately, we were unable to perform these measurements in the current study.

As for other dietary fibers, supplementing the diet of CKD patients with arabinoxylan oligosaccharide (20 g/day) also had no effect on serum levels or 24-hour urinary excretion rates of IS while supplementing the diet of HD patients with high-amylose corn starch (15 g/day) reduced free IS levels [14, 15].

In this trial, GA supplementation to CKD patients was associated with a significant reduction in CRP levels. This effect was evident in patients who received only 10 g/day of GA, supporting previous observations that 10 g/day of GA provided an optimal prebiotic effect [8]. As far as we know, this is the first trial to demonstrate that dietary fiber supplementation can reduce inflammatory markers in predialysis CKD patients. Previous trials of dietary fiber supplementation to predialysis CKD patients found no effect on CRP level, including trials that used arabinoxylan oligosaccharide and combined pea hull and inulin [14, 16]. Similarly negative results were obtained after supplementing the diet of HD patients with high-amylose corn starch or oligofructose-enriched inulin [15, 17]. The only trial that found a positive effect of dietary fiber supplementation on CRP level was conducted on a group of HD patients utilizing 10–20 g/day of a highly fermentable dietary fiber (type not specified). The authors reported significantly reduced CRP (10.7 ± 4.8 to 4.8 ± 4.5 mg/L, *p* < 0.05), IL-6, and TNF-*α* levels after dietary fiber supplementation [19].

Because of the tertiary care nature of our hospital, many patients under regular follow-up in the nephrology clinic were excluded from this trial because of malignancy or liver disease. Other patients declined participation because they were dependent on family members for transportation and wanted to avoid an extra visit to the hospital. This severely restricted the number of participants. Consequently, we were unable to include a control group and had to rely on the paired *t*-test to confirm the statistical significance of our observations. Additionally, we were unable to perform stool bacterial counts or measure 24-hour urinary excretion rates of IS as an indirect indicator of protein-fermenting bacteria in the colon.

Despite these limitations, we were able to demonstrate for the first time that GA supplementation reduces inflammatory markers in predialysis CKD patients. CRP may be a nonspecific marker of inflammation, but it has been shown in epidemiological studies to be inversely related to mortality in CKD patients [6]. In our opinion, this trial lays the ground for a large, randomized, and controlled clinical trial to determine the long-term effect of GA supplementation on CKD mortality and disease progression.

## 5. Conclusions

Supplementing the diet of CKD patients with 10–40 g/day of GA had no demonstrable effect on BUN and IS levels. Nevertheless, GA supplementation was associated with a significant reduction in CRP level. This could have a positive impact on the long-term morbidity and mortality of this patient population.

## Figures and Tables

**Table 1:** Baseline characteristics of the study population (*n* = 30).

Parameter	Group-1 (10 g/day)	Group-2 (20 g/day)	Group-3 (40 g/day)	*p* value
Number	12	9	9	
Age	48 ± 12 (30–66)	52 ± 18 (18–75)	50 ± 17 (18–74)	0.8
Male/female	8/4	6/3	6/3	1.0
Diabetes mellitus	41.7%	33.3%	55.6%	0.6
CKD stage 3B/4	8/4	3/6	4/5	0.3
eGFR (mL/min/1.7 m^2^)	26 ± 10	33 ± 11	29 ± 9	0.3
Creatinine (*µ*mol/L)	250 ± 92	201 ± 78	216 ± 48	0.3
BUN (mmol/L)	14 ± 6	12 ± 5	14 ± 4	0.4
Immunosuppression^*∗*^	33.3%	22.2%	22.2%	0.8

CKD: chronic kidney disease; eGFR: estimated glomerular filtration rate; BUN: blood urea nitrogen.

^*∗*^For systemic lupus erythematosus and chronic glomerulonephritis.

**Table 2:** Effect of Gum Arabic supplementation on renal parameters (*n* = 30).

Parameter	Baseline level	Follow-up level	*p* value
eGFR (mL/min/1.7 m^2^)	29.1 ± 9.9	29.5 ± 11.1	0.5
Creatinine (*µ*mol/L)	225 ± 77	227 ± 83	0.7
BUN (mmol/L)	13.5 ± 5.0	13.6 ± 5.3	0.8
Sodium (mmol/L)	137.8 ± 2.4 (131–143)	136.3 ± 3.1 (128–141)	0.002^*∗*^
Potassium (mmol/L)	4.5 ± 0.5	4.6 ± 0.6	0.3
Bicarbonate (mmol/L)	24.0 ± 3.7	24.2 ± 3.2	0.8
Uric acid (mmol/L)	466 ± 98	453 ± 96	0.3
Calcium (mmol/L)	2.4 ± 0.1	2.4 ± 0.1	0.1
Phosphorus (mmol/L)	1.2 ± 0.2	1.3 ± 0.2	0.8
Magnesium (mmol/L)	0.8 ± 0.2	0.8 ± 0.1	0.9
Parathyroid hormone (pg/mL)	256 ± 334	256 ± 316	1.0
Hemoglobin (g/dL)	12.1 ± 2.5	12.1 ± 2.3	0.8
Indoxyl sulfate (mg/L)	1.2 ± 2.9	1.2 ± 2.4	0.9
Urine volume (mL/day)	2116 ± 704	2167 ± 692	0.6
Creatinine excretion (g/day)	1.1 ± 0.4	1.1 ± 0.4	0.6
Urea excretion (mmol/day)	268 ± 88	275 ± 89	0.7
Protein excretion (g/day)	3.2 ± 4.1	3.4 ± 4.8	0.6
Creatinine clearance (mL/day)	33.4 ± 14.4	34.8 ± 17.4	0.4
Urea clearance (mL/day)	15.6 ± 7.7	16.3 ± 8.5	0.4

eGFR: estimated glomerular filtration rate; BUN: blood urea nitrogen.

^*∗*^Statistically significant.

**Table 3:** Effect of Gum Arabic dietary supplementation on mean values of various inflammatory markers in studied chronic kidney disease patients (*n* = 30).

Parameter	Baseline	Follow-up	*p* value
Serum CRP (ng/mL)	3.5 ± 1.5	2.8 ± 1.6	0.02^*∗*^
Serum IL-1 (pg/mL)	0.39 ± 0.38	0.35 ± 0.41	0.7
Serum IL-2 (pg/mL)	26.0 ± 128	17.2 ± 82	0.3
Serum IL-4 (pg/mL)	0.24 ± 0.15	1.37 ± 6.1	0.3
Serum IL-5 (pg/mL)	36.9 ± 137	15.5 ± 45.5	0.2
Serum IL-6 (pg/mL)	29.9 ± 80.1	20.9 ± 47.9	0.5
Serum IL-10 (pg/mL)	3.9 ± 2.9	3.3 ± 2.9	0.3
Serum IL-12 (pg/mL)	1.0 ± 3.7	0.5 ± 1.4	0.3
Serum IL-13 (pg/mL)	0.4 ± 0.4	0.3 ± 0.4	0.3
Serum IFN-*γ* (pg/mL)	0.93 ± 0.93	2.1 ± 7.6	0.4
Serum TNF-*α* (pg/mL)	0.58 ± 0.6	0.37 ± 0.5	0.2
Urinary TGF-*β* (pg/mL)	14.8 ± 16.3	17.7 ± 19.9	0.4

CRP: C-reactive protein; IL: interleukin; IFN: interferon; TNF: tumor necrosis factor; TGF: transforming growth factor.

^*∗*^Statistically significant.

**Table 4:** The severity of gastrointestinal symptoms at baseline and at follow-up among studied chronic kidney disease patients (*n* = 30).

Symptom	Baseline (%)			Follow-up (%)			*p* value
	None	Mild	Severe	None	Mild	Severe	
Abdominal pain	70	30	0	73.3	23.3	3.3	1
Abdominal distention	60	40	0	73.3	23.3	3.3	0.2
Flatulence	60	36.7	3.3	53.3	26.7	20	0.1
Nausea	93.3	6.7	0	93.3	6.7	0	1
Diarrhea	90	10	0	93.3	6.7	0	0.7
Constipation	63.3	26.7	10	73.3	23.3	3.3	0.3
